# A Novel Ultra‐Sensitive Semiconductor SERS Substrate Boosted by the Coupled Resonance Effect

**DOI:** 10.1002/advs.201900310

**Published:** 2019-04-16

**Authors:** Lili Yang, Yusi Peng, Yong Yang, Jianjun Liu, Haoliang Huang, Bohan Yu, Jimin Zhao, Yalin Lu, Zhengren Huang, Zhiyuan Li, John R. Lombardi

**Affiliations:** ^1^ State Key Laboratory of High Performance Ceramics and Superfine Microstructures Shanghai Institute of Ceramics Chinese Academy of Sciences 1295 Dingxi Road Shanghai 200050 P. R. China; ^2^ University of Chinese Academy of Sciences No.19(A) Yuquan Road Beijing 100049 P. R. China; ^3^ Center of Materials Science and Optoelectronics Engineering University of Chinese Academy of Sciences Beijing 100049 P. R. China; ^4^ National Synchrotron Radiation Laboratory University of Science and Technology of China Hefei 230026 P. R. China; ^5^ Beijing National Laboratory for Condensed Matter Physics Institute of Physics Chinese Academy of Sciences Beijing 100190 P. R. China; ^6^ South China University of Technology Guangzhou 510640 Guangdong P. R. China; ^7^ Department of Chemistry The City College of New York 160 Convent Avenue New York NY 10031 USA

**Keywords:** energy band engineering, photoinduced degradation, surface‐enhanced Raman scattering, the “coupled resonance” effect, ultra‐sensitive Ta_2_O_5_ nanorod substrate

## Abstract

Recent achievements in semiconductor surface‐enhanced Raman scattering (SERS) substrates have greatly expanded the application of SERS technique in various fields. However, exploring novel ultra‐sensitive semiconductor SERS materials is a high‐priority task. Here, a new semiconductor SERS‐active substrate, Ta_2_O_5_, is developed and an important strategy, the “coupled resonance” effect, is presented, to optimize the SERS performance of semiconductor materials by energy band engineering. The optimized Mo‐doped Ta_2_O_5_ substrate exhibits a remarkable SERS sensitivity with an enhancement factor of 2.2 × 10^7^ and a very low detection limit of 9 × 10^−9^ m for methyl violet (MV) molecules, demonstrating one of the highest sensitivities among those reported for semiconductor SERS substrates. This remarkable enhancement can be attributed to the synergistic resonance enhancement of three components under 532 nm laser excitation: i) MV molecular resonance, ii) photoinduced charge transfer resonance between MV molecules and Ta_2_O_5_ nanorods, and iii) electromagnetic enhancement around the “gap” and “tip” of anisotropic Ta_2_O_5_ nanorods. Furthermore, it is discovered that the concomitant photoinduced degradation of the probed molecules in the time‐scale of SERS detection is a non‐negligible factor that limits the SERS performance of semiconductors with photocatalytic activity.

## 1 Introduction

Surface‐enhanced Raman scattering (SERS) is an extremely sensitive and practical analytical technology that is able to enhance the Raman signal of molecules absorbed on special substrates' surfaces by orders of magnitude.^1^, ^2^ Its sensitive identification ability for chemical and biological molecules promotes its widespread application in numerous fields such as biological, pharmaceutical, contaminant, and toxin detection.^3^, ^4^, ^5^, ^6^ The SERS effect can be well expressed by optimized SERS substrate materials, which can produce a tremendous enhancement effect through two dominant mechanisms: the electromagnetic mechanism (EM) and the chemical mechanism (CM).^7^, ^8^ Among these mechanisms, the EM takes effect when there is a strong local electromagnetic field enhancement induced by either the localized surface plasmon resonance (LSPR) excited by the excitation light or through Mie scattering resonances, while the CM is believed to be mainly based on the resonance of the charge transfer (CT) process occurring in the complexes of substrate materials and absorbed molecules with the incident light.^9^, ^10^ Noble metals such as Au, Ag, and Cu have become some of the most popular SERS substrate materials with an enhancement factor (EF) up to the order of 10^14^ due to the foremost contribution of the EM.^4^, ^11^ However, their developments are often limited by their increasingly exposed shortcomings, such as the low number of varieties, poor biocompatibility, easy agglomeration, secondary pollution, and nonreusability. Compared with the first discovery of noble metals' SERS effect in 1974,^12^ research on the semiconductor SERS substrates, in which the CM has conversely been revealed to play a major role, was launched later. Although their enhancement effect is usually low, between 10–10^5^, and the problem of their unclear and controversial enhancement mechanism has persisted, semiconductors can still become one of the most promising candidate SERS substrate materials due to their rich variety, excellent biocompatibility, and useful versatility.^13^, ^14^ Additionally, the high selectivity of semiconductor SERS substrates to probed molecules can also become a unique advantage when distinguishing a specific molecule from the complex environment with many other molecules.

Currently, the objectives receiving the most focus regarding semiconductor SERS substrates are improving their SERS performance and exploring their intrinsic enhancement mechanism, and the two complement each other. As an increasing number of researchers are engaging in the study of semiconductor SERS substrates, research achievements regarding semiconductor SERS substrates have grown exponentially in recent years. From porous ZnO nanosheets,^15^ TiO_2_ photonic microarrays,^16^ CuTe quantum dots,^17^ chemically etched ZnSe,^18^ and single Cu_2_O superstructure particles^19^ to urchin‐like W_18_O_49_,^20^ MoO_3−_ *_x_*@MoO_3_ nanosheets,^21^ MoO_3−_ *_x_* quantum dots,^22^ and oxygen‐incorporating MoS*_x_*O*_y_*,^23^ two types of methods are generally used to improve the SERS performance of semiconductor substrates: morphology design and element doping. Quantifying the size of nanocrystals and doping heavily with other elements in semiconductors can greatly increase the carrier concentration and shift the LSPR peak to the near‐infrared or visible region accordingly,^24^ thus resulting in some metal‐like properties and improved SERS activity in semiconductors. However, recent research on semiconductor SERS substrates has encountered a bottleneck. For many conventional semiconductor SERS substrates, even though they are shaped variously, or they are doped using a variety of methods, including self‐doping and hetero‐doping, it is still difficult for them to exceed an EF of 10^6^. Therefore, exploring a new type of semiconductor that has high SERS performance and that features further enhanced SERS activity to a limit is a high‐priority task.

Usually, metal nanoparticles exhibit great SERS enhancement, which is mainly attributed to EM enhancement because EM enhancement is several orders of magnitude stronger than CM enhancement. However, whether EM‐SERS enhancement or CM‐SERS enhancement (including molecular resonance and CT resonance) is operative in reported semiconductor substrates, the individual contribution for SERS is usually less than 10^5^. To obtain the highest SERS enhancement in semiconductor substrates, the semiconductor‐molecule system and experimental parameters must be carefully selected to match the SERS selection rules and produce cooperative resonance effects, including molecular resonance, molecule‐semiconductor CT resonance, and electromagnetic resonance including metal‐like surface plasmon resonance and Mie scattering resonance of semiconductors under an excitation laser. Therefore, we present a “coupled resonance” strategy for optimizing semiconductor SERS sensitivity in order to fulfill three quasi‐resonance Raman effects, molecular resonance λ_mol_, molecule‐semiconductor CT resonance λ_CT_, and semiconductor electromagnetic resonance λ_EM_ including plasmon or Mie scattering resonance,^25^ at or near the laser excitation wavelength λ_laser_ via a multicomponent design including bandgap engineering and morphology tuning. We invoke a quasi‐approximation here that is given as follows

(1) $$\lambda_{\mathrm{mol}}\approx \lambda_{\mathrm{CT}}\approx \lambda_{\mathrm{EM}}\approx \lambda_{\mathrm{laser}}$$

**Scheme 1:**
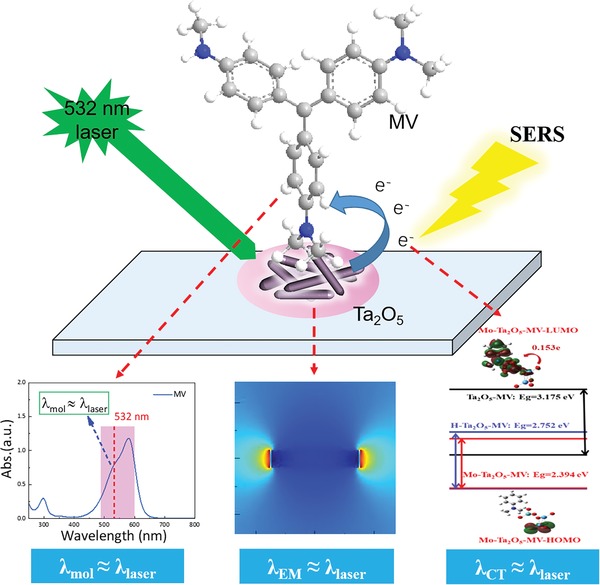
Mechanism diagram of the “coupled resonance” strategy on the semiconductor Ta_2_O_5_ SERS substrate.

Among the factors in Equation (1), the favorable CT and electromagnetic resonances are expected to be regulated to quasi‐match the resonance frequency of the given incident laser and the carefully selected molecule via energy engineering. All three resonances are chosen to be synergistically realized in novel semiconductor substrates (**Scheme** **1**).

As a wide bandgap semiconductor, Ta_2_O_5_ has many excellent properties, such as a high refractive index, excellent photoelectric performance, and high chemical stability.^26^, ^27^ In addition, it has also been applied in the field of photocatalysis due to its suitable conduction band (CB) and valence band (VB) positions.^28^ However, the SERS property of tantalum‐based oxide (Ta_2_O_5_) has never been reported. At the same time, with the discovery of the intrinsic outstanding SERS performance of Nb_2_O_5_,^29^ we expect ultra‐high SERS and photocatalytic self‐cleaning performance to be synergistically realized in Ta_2_O_5_, in which the element Ta is located in the same VB family as the element Nb.

In this work, Ta_2_O_5_ nanorods (NRs) were first synthesized, and then energy band engineering through element doping (hydrogen reduction and high‐valence Mo^6+^ doping) was adopted to further improve the SERS performance. The Mo‐doped Ta_2_O_5_ substrate exhibited a remarkable SERS sensitivity with an EF of 2.2 × 10^7^ and a very low detection limit of 9 × 10^−9^ m for methyl violet (MV), which is better than those of most reported semiconductor SERS substrates. The synergistic resonance enhancement mechanism of three components under 532 nm laser excitation, namely, i) MV molecular resonance, ii) photoinduced charge transfer (PICT) resonance between MV molecules and Ta_2_O_5_ NRs, and iii) EM enhancement around the “gap” and “tip” of anisotropic Ta_2_O_5_ NRs were demonstrated in our novel Mo‐doped Ta_2_O_5_ NRs by experiments and first‐principles calculations. This work offers a cost‐effective pathway to design novel ultra‐sensitive semiconductor SERS‐substrates.

Simultaneously, along with the synergistic realization of the good photocatalytic self‐cleaning capability of the ultra‐sensitive Mo‐Ta_2_O_5_ SERS substrates, we discovered the concomitant photoinduced degradation of the probed molecules on the Ta_2_O_5_ substrates during the process of SERS detection by ultrafast spectroscopy. In addition to the well‐known phenomenon of photobleaching of dye molecules in metal SERS materials by strong lasers,^30^ here the unique photocatalytic degradation of probed molecules by semiconductors was revealed to be another non‐negligible factor that limits the SERS performance of some semiconductors with photocatalytic activity in SERS measurement.

## 2 Results

### 2.1 Sample Characterizations

**Figure 1:**
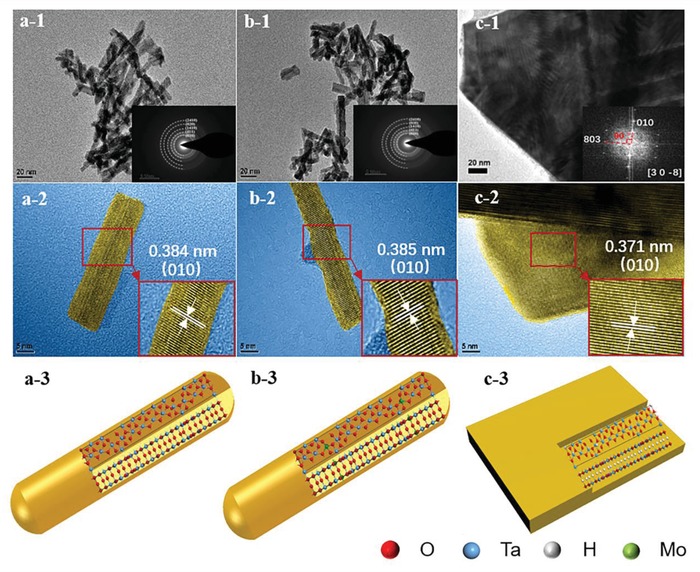
TEM (1), HRTEM (2) images, and schematic diagram for crystal structures (3) of a) Ta_2_O_5_ NRs, b) 15%‐Mo‐Ta_2_O_5_ NRs, and c) H‐Ta_2_O_5_ NSs substrates. Inset in (a‐1): SAED pattern of Ta_2_O_5_ NRs. Inset in (b‐1): SAED pattern of 15%‐Mo‐Ta_2_O_5_ NRs. Inset in (c‐1): Fast Fourier transform (FFT) of framed area in (c‐2). Insets in (a‐2,b‐2,c‐2) show partial enlarged details of the respective images.

**Figure 2:**
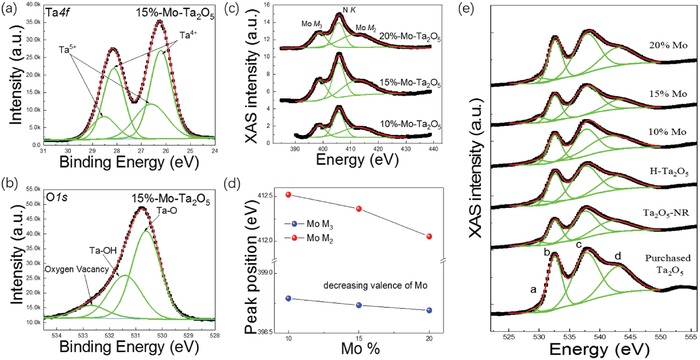
a) Ta4f XPS spectrum of 15%‐Mo‐Ta_2_O_5_ NRs substrates and b) O1s XPS spectrum of 15%‐Mo‐Ta_2_O_5_ NRs substrates. c) Mo M‐edge XAS spectra of the *x*‐Mo‐Ta_2_O_5_ samples (*x* = 10%, 15%, and 20%) and d) the corresponding valence change of element Mo. e) O K‐edge XAS spectra of the purchased Ta_2_O_5_, Ta_2_O_5_ NR, H‐Ta_2_O_5_, and *x*‐Mo‐Ta_2_O_5_ samples (*x* = 10%, 15%, and 20%).

The shape‐controlled Ta_2_O_5_ NRs and Mo‐doped Ta_2_O_5_ NRs were first obtained by an improved hydrothermal reaction according to the literature,^27^ followed by hydrogenation on the Ta_2_O_5_ NRs to derive hydrogenated Ta_2_O_5_ nanosheets (H‐Ta_2_O_5_ NSs). Transmission electron microscopy (TEM) and high‐resolution TEM (HRTEM) were used to study the morphological evolution and crystal structures of different Ta_2_O_5_ SERS substrates (**Figure** 1). The derived Ta_2_O_5_ NRs possessed a diameter of ≈5–10 nm, whereas the length was in the range of 20–50 nm (Figure 1a‐1), and the clear lattice fringes on single Ta_2_O_5_ NRs can be easily discerned by HRTEM (Figure 1a‐2) to correspond to the (010) planes of orthorhombic Ta_2_O_5_ (JCPDS. 79‐1375) with an inter‐planar spacing of 0.384 nm. This finding indicates that the Ta_2_O_5_ NRs preferably grew along the [010] direction. It can be clearly inferred from Figure 1b‐1 that the Mo‐doped Ta_2_O_5_ (Mo‐Ta_2_O_5_) still maintains the NR nanostructure after element doping. As the concentration of Mo added to the precursor increased, the nanorods exhibited an obvious increased length, whereas there were no significant changes in diameter. The average lengths were 25.5, 35.8, and 42.7 nm for the 10%‐, 15%‐, and 20%‐Mo‐Ta_2_O_5_ substrates, respectively (Figure S1, Supporting Information). However, the low‐valence hydrogen‐reduced Ta_2_O_5_ exhibited a very different appearance. After annealing in Ar/H_2_ and deoxygenating over the H‐Ta_2_O_5_ substrate, the Ta_2_O_5_ NRs were completely converted to well‐crystallized H‐Ta_2_O_5_ NSs (Figure 1c‐1); Figure 1c‐2 shows the corresponding HRTEM pattern of the nanosheets taken along the [30‐8] zone axis (inset in Figure 1c‐1). This transformation can be explained by the crystal structure model of Ta_2_O_5_ NRs, in which the interlayer oxygen atoms were easily destroyed by hydrogen atoms and the Ta_2_O_5_ NRs were finally transformed into Ta_2_O_5_ nanosheets. The phase and crystallinity of the samples were also analyzed by X‐ray diffraction (XRD). As shown in Figure S2 (Supporting Information), the Ta_2_O_5_ NRs were crystallized with Ta_2_O_5_ (JCPDS. 79‐1375) phases with lattice constants corresponding to the orthorhombic Ta_2_O_5_ structure with space group *Pmm*2 (*a* = 43.997 Å, *b* = 3.894 Å, and *c* = 6.209 Å). The strongest peak indexed to (010) suggested a preferential growth along the *b* axis, whereas other crystal planes, namely, (411), (1410), (020), and (2410), in Ta_2_O_5_ exhibited widened peaks due to the weaker crystallization; these crystal planes were highly aligned with the polycrystalline ring presented in the selected area electron diffraction (SAED) pattern of the Ta_2_O_5_ substrates (insets of Figure 1a‐1) in addition to the HRTEM pattern. When investigating the crystal structure of Mo‐doped Ta_2_O_5_, it can be found from Figure S2 (Supporting Information) that (010) was still the strongest peak and that there was no significant shift in the XRD peak positions as the incorporation content of Mo increased, revealing that Mo was uniformly dissolved into Ta_2_O_5_ due to the similar ionic radius of Mo (0.65 Å) and Ta (0.64 Å).

X‐ray photoelectron spectroscopy (XPS) was further used to obtain more detailed information about the superficial chemical state of the doped substrates. Ta and O can be clearly identified in the XPS spectra of both the H‐Ta_2_O_5_ and Mo‐doped Ta_2_O_5_, among which both of the Ta4f peaks presented an obvious displacement to lower binding energies (Figure S3a, Supporting Information), whereas XPS analysis of O1s exhibited a significant shift to a higher binding energy (Figure S3b, Supporting Information), indicating that some of the Ta^5+^ in both samples was reduced to a lower valence state. Figure S3c (Supporting Information) shows the high‐resolution XPS spectra of Ta4f in the H‐Ta_2_O_5_ sample. In addition to doublet peaks arising from Ta^5+^, there were two more unambiguous doublet peaks located at 28.17 eV (Ta4f_5/2_) and 26.25 eV (Ta4f_7/2_), which belonged to Ta^4+^ and suggested that reduction occurred during hydrogenation.^31^, ^32^ Furthermore, the enlarged characteristic peaks in the O1s region located at 531.4 and 532.7 eV were attributed to the Ta‐OH and oxygen vacancy (*V*_O_), respectively (Figure S3d, Supporting Information), thus confirming the emergence of Ta^4+^ and the damage to the interlayer oxygen atoms in the H‐Ta_2_O_5_ NSs by hydrogen atoms (please refer to Figure S5c‐1 in the Supporting Information for more details). A similar reduction of Ta^5+^ to Ta^4+^ and the appearance of *V*_O_ can also be observed in the Ta4f and O1s XPS spectra of Mo‐doped Ta_2_O_5_ (**Figure** 2a,b). This was mainly because the local electron cloud density around the six‐coordinated Ta increased after the easier substitution of the nearby four‐coordinated Ta with a large surrounding space by higher‐valence Mo (please refer to Figure S5b‐1 in the Supporting Information for more details).

To clarify the behavior of the Mo dopants and the local structures of Mo and Ta atoms in Mo‐Ta_2_O_5_, X‐ray absorption spectroscopy (XAS) was adopted to detect their chemical states. Although the Mo3d peaks were completely submerged in the Ta4d peaks in the XPS spectra, the M_2_‐edge and M_3_‐edge of Mo in the XAS spectra (Figure 2c) can verify the presence of Mo in the Mo‐doped Ta_2_O_5_ substrates.^33^, ^34^, ^35^ After analyzing the peak position of Mo M_2_ and Mo M_3_, it can easily be observed that the valence of Mo decreased with increasing Mo content (Figure 2d). Fundamentally, regardless of the high‐valence Mo^6+^ doping or the low‐valence hydrogen reduction, some of the local Ta^5+^ was reduced to lower‐valence Ta^4+^ accompanied by the appearance of *V*_O_. Subsequently, O K‐edge XAS spectra of the purchased Ta_2_O_5_, Ta_2_O_5_ NR, H‐Ta_2_O_5_, and Mo‐Ta_2_O_5_ samples were further analyzed by fitting with peaks a, b, c, and d (Figure 2e), which were located at 529.2, 532.6, 537.8, and 543.1 eV, respectively. Peak a was assigned to the transition from oxygen vacancies or oxygen holes, and peaks b, c and d arise from the transitions of O 1s to O2p‐Ta5d (*t*_2g_)/Mo4d (*t*_2g_), O2p‐Ta5d (*e*_2g_)/Mo4d (*e*_2g_), and O2p‐Ta6sp, respectively.^36^, ^37^, ^38^ By comparison among these Ta_2_O_5_ substrates, peak a in the 15%‐Mo‐Ta_2_O_5_ and H‐Ta_2_O_5_ substrates was found to be relatively obvious, indicating the appearance of more oxygen vacancies or oxygen holes due to the high‐valence Mo^6+^ doping and the low‐valence hydrogen reduction (Table S1, Supporting Information). Under the same measurement conditions, the full width at half maximum (FWHM) of peak d was decreased followed by an increase with the increase of incorporated Mo^6+^ concentration (Table S1, Supporting Information). It can be attributed to that different doping concentration produce different orbital hybridization, and there are less core holes at the O 2p‐Ta 6sp energy level for 15%‐Mo‐Ta_2_O_5_.

### 2.2 Raman Enhancement for the Ta_2_O_5_ Sample

**Figure 3:**
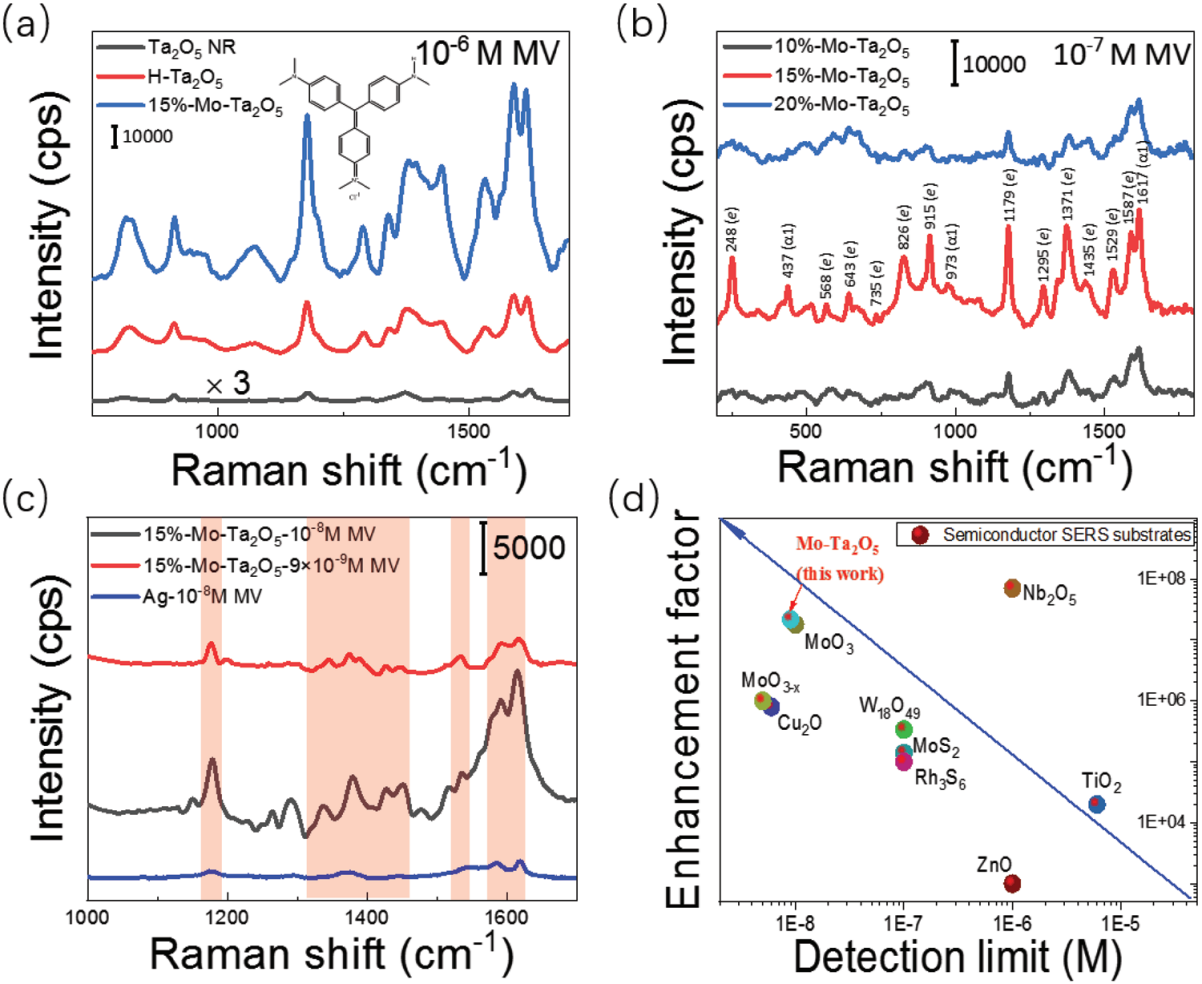
a) SERS spectra of 10^−6^ m MV on Ta_2_O_5_ NRs, H‐Ta_2_O_5_ NSs, and 15%‐Mo‐Ta_2_O_5_ substrates. b) SERS spectra of 10^−7^ m MV on *x*‐Mo‐Ta_2_O_5_ substrates (*x* = 10%, 15%, and 20%). c) Exploring the detection limits of 15%‐Mo‐Ta_2_O_5_ substrate and comparing SERS performance of our substrates with noble metal Ag without “hot spot” effect. d) Comparison of SERS performance of different reported semiconductor nanostructure substrates.

We were mainly concerned with the effects of the energy band adjustment through element doping (high‐valence Mo^6+^ doping and low‐valence hydrogen reduction) on the SERS performance of Ta_2_O_5_ substrates. The dye MV was used as a Raman probe to examine the Raman enhancement behavior of the Ta_2_O_5_ substrates because MV exhibited a strong optical absorption peak and molecular resonance near the laser excitation wavelength of 532 nm (λ_mol_ ≈ λ_laser_). The intrinsic Raman spectra of MV and substrates are shown in Figure S4 (Supporting Information). Interestingly, the hydrogenated Ta_2_O_5_ substrate exhibited a much better Raman enhancement ability than the Ta_2_O_5_ NR substrates when detecting 10^−6^ m MV, whereas its SERS behavior was regretfully much inferior to that of the 15%‐Mo‐Ta_2_O_5_ substrate (**Figure** 3a). SERS spectra of 10^−7^ m MV under the irradiation with a 532 nm laser on Ta_2_O_5_ substrates doped with different amounts of Mo are shown in Figure 3b. The three main SERS peaks of MV on our 15%‐Mo‐Ta_2_O_5_ substrates located at 1617 cm^−1^ (the totally symmetric 8a benzene mode), 1371 (phenyl‐*N* stretching, asymmetric stretching of the central C—C—C bonds, bending of the ring C—C—C and C—H bonds), and 1179 cm^−1^ (asymmetric 9a benzene mode, asymmetric stretching of the central C—C—C bonds). All these peaks and the other observed SERS peaks, which are listed in Table S2 (Supporting Information), agreed well with the characteristic peaks of MV.^39^ As anticipated, when we gradually incorporated high‐valence Mo^6+^ into the Ta_2_O_5_ NR substrate, its SERS performance obviously became superior to that of the pure Ta_2_O_5_ NRs. Moreover, as the amount of Mo incorporated gradually increased, the SERS signal of 10^−7^ m MV first exhibited an improvement followed by an attenuation. This may be due to the most suitable doping concentration to obtain the best energy band structural match between MV molecules and the doped Ta_2_O_5_. Among the concentrations evaluated, the optimal doping concentration of Mo was 15%, and it can be found from Figure 3c that compared to the precious metal Ag without “hot spots,” this superfine 15%‐Mo‐Ta_2_O_5_ SERS substrate can also exhibit better performance when detecting 10^−8^ m MV. Moreover, even when the concentration of MV aqueous solution was diluted to 9 × 10^−9^ m, the Raman lines of MV molecules on our 15%‐Mo‐Ta_2_O_5_ substrate at 1617, 1587, 1529, 1371, and 1179 cm^−1^ could still be conspicuously identified despite the gradually apparent Raman signal background from the substrate (Figure 3c). Encouragingly, our substrates possessed a very low detection limit of 9 × 10^−9^ m, and the corresponding EF^40^ was determined to be 2.2 × 10^7^ with the Raman line at 1617 cm^−1^ (calculation details in the Supporting Information 2). To the best of our knowledge, this SERS performance is one of the highest sensitivities among those reported for semiconductor SERS substrates (as shown in Figure 3d and Table S3, Supporting Information) under a laser excitation wavelength of 532 nm and even parallels that of the noble metals without “hot spots.”

### 2.3 The SERS Enhancement Mechanism of Ta_2_O_5_ Substrates by Different Element Doping

According to the “Unified View” presented by Lombardi et al.,^13^, ^41^, ^42^ the polarizability involving an infinite sum over all the molecule states *R*_mol−CT_(ω) can be introduced to evaluate the surface‐enhanced Raman intensity, which is proportional to |*R*_mol−CT_(ω)|^2^ for a single dominant term. *R*_mol−CT_(ω) can be expressed as

(2) $$R_{\mathrm{mol‐CT}}\left(\omega \right)=\frac{\left[\left(\mu_{\mathrm{mol}}^{\alpha }E^{\alpha }\right)\left(\mu_{\mathrm{CT}}^{\beta }E^{\beta }\right)h_{\mathrm{mol‐CT}}〈i\left|Q_{K}\right|f〉\right]}{\left[{\left(\varepsilon_{1}\left(\omega \right)+2\varepsilon_{0}\right)}^{2}+\chi \varepsilon_{2^{}}{}^{2}\right]\left[\left(\omega_{}^{2}{}_{\mathrm{CT}}-\omega^{2}\right)+\gamma_{\mathrm{CT}}{}^{2}\right]\left[\left(\omega_{}^{2}{}_{\mathrm{mol}}-\omega^{2}\right)+\gamma_{}^{2}{}_{\mathrm{mol}}\right]}$$

In the expression, *E* ^α,β^ represents the electric field at the surface (in the a, b = *x*, *y*, z direction) of the substrates due either to the surface plasmon or the Mie scattering resonance, and *Q*_K_ is the molecular normal mode. The molecular resonance (μ_mol_) and the CT resonance (μ_CT_) are coupled to each other through the Herzberg–Teller coupling constant (*h*_mol−CT_). ε_1_and ε_2_ refer to the real and imaginary parts, respectively, of the substrates' dielectric constant and ε_0_ is the real part of the dielectric constant of the surrounding medium. χ is a constant factor that accounts for the geometry of the substrate if it deviates from a spherical shape. From Formula (2), there are three parts in the denominator jointly contributing to the SERS intensity. When the incident light frequency coincides with a certain resonance frequency of the system, the corresponding portion must be involved in the SERS enhancement. The EM resonance dominates when ε_1_ (ω) = −2ε_0_. When ω  = ω_CT_, there is CT resonance involving the transfer of electrons between the molecules and the substrates. Accordingly, ω  = ω_mol_ indicates molecular resonance. Thus, one or more resonances will play a leading role when given an incident laser with a fixed frequency. Which one, two or three of these specific resonances will participate depends on the specific molecular and substrate parameters. Usually, nontotally symmetric vibration in addition to totally symmetric modes are excited through Herzberg–Teller vibronic coupling,^43^ which intimately links the above three resonances. The totally symmetric a_1_ vibration modes tend to dominate the spectrum when only surface plasmon resonances contribute through the Franck–Condon term.^44^, ^45^ It can be observed from Figure 3b that only the lines at 1617, 973, and 437 cm^−1^ can be ascribed to the totally symmetric a_1_ vibrations, whereas many other lines at 1587, 1529, 1435, 1371, 1295, 1179, 915, and 826 cm^−1^ can be assigned to the nontotally symmetric e modes.^39^ Apparently, the SERS spectra of MV on our Ta_2_O_5_ substrates were considerably dominated by the nontotally symmetric e vibrations, indicating a dominant CT or molecular resonances in this case. As previously discussed, both high‐valence Mo^6+^ doping and low‐valence hydrogen reduction resulted in the appearance of lower‐valence Ta^4+^ and *V*_O_. The SERS performance discrepancy among these doped Ta_2_O_5_ substrates was most likely caused by the different resonance intensities induced by the CT between the substrates and probed molecules under the incident laser.

**Figure 4:**
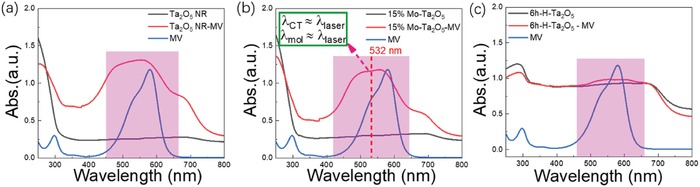
a) UV–vis optical absorption spectrum of Ta_2_O_5_ powders, MV modified Mo‐Ta_2_O_5_ powders and MV aqueous solution. b) UV–vis optical absorption spectrum of 15%‐Mo‐Ta_2_O_5_ powders, MV modified 15%‐Mo‐Ta_2_O_5_ powders and MV aqueous solution. c) UV–vis optical absorption spectrum of H‐Ta_2_O_5_ powders, MV modified H‐Ta_2_O_5_ powders and MV aqueous solution.

We first analyzed the contribution of the CM (including MV molecular resonance and CT resonance) to the SERS performance of these Ta_2_O_5_ substrates. **Figure** 4 shows the UV–vis optical absorption spectrum of Ta_2_O_5_ NRs, 15%‐Mo‐Ta_2_O_5_ powder and H‐Ta_2_O_5_ powder with/without MV absorption. The probed molecule MV exhibited a strong optical absorption peak and molecular resonance (λ_mol_) near the laser excitation wavelength of 532 nm, indicating an intrinsic resonance frequency matching with the 532 nm incident laser as follows

(3) $$\lambda_{\mathrm{mol}}\approx \lambda_{\mathrm{laser}}$$

After the probed MV molecules were adsorbed on the surface of the 15%‐Mo‐Ta_2_O_5_ and H‐Ta_2_O_5_ substrates, it can be clearly identified that the absorption peaks of the MV all presented certain degrees of blueshift and broadening, revealing a strong chemical interaction and CT process between the MV and our Ta_2_O_5_ substrates.^46^ The broadened absorption peaks of the MV‐Ta_2_O_5_ complexes were located in the wavelength region of 500–600 nm and indicated CT resonance in this range, which was very close to the excitation wavelength (532 nm). Thus, the quasi‐resonance λ_CT_ ≈ λ_laser_ was tenable here and indicated the dominance of the CT process in the surface‐enhanced Raman spectrum.

To further analyze the effect of the different element doping strategies in energy band engineering on the SERS performance of Ta_2_O_5_ substrates, Ta_2_O_5_, Mo‐Ta_2_O_5_, and H‐Ta_2_O_5_ models were constructed to investigate the specific degree of charge‐transfer in these three complex systems. Incorporation of Mo into the Ta_2_O_5_ NRs had two structurally different types: preferential occupation of the Mo atoms in the large lattice gap site of Ta_2_O_5_ or substitution of the Ta atom on the lattice position with Mo atoms. According to the XRD results, which showed no obvious XRD peak displacement of Ta_2_O_5_ after Mo doping, the incorporated Mo^6+^ was most likely in the mode of substituting Ta^5+^ due to the similar ion radius of Mo^6+^ (0.65 Å) and Ta^5+^ (0.64 Å). Therefore, we constructed a crystal structure of Ta_2_O_5_ NRs with a space group of *Pmm*2 according to the XRD results (Figure S5a‐1, Supporting Information), and then replaced the Ta atom with a larger Mo atom to obtain a crystal structural model of Mo‐Ta_2_O_5_ (Figure S5b‐1, Supporting Information). Similarly, according to the formation of NSs observed in TEM analysis and the appearance of oxygen vacancies in XPS analysis, the H‐Ta_2_O_5_ crystal structure was constructed by substituting the interlayer oxygen atoms with H atoms to form the H‐Ta_2_O_5_ model (Figure S5c‐1, Supporting Information).

**Figure 5:**
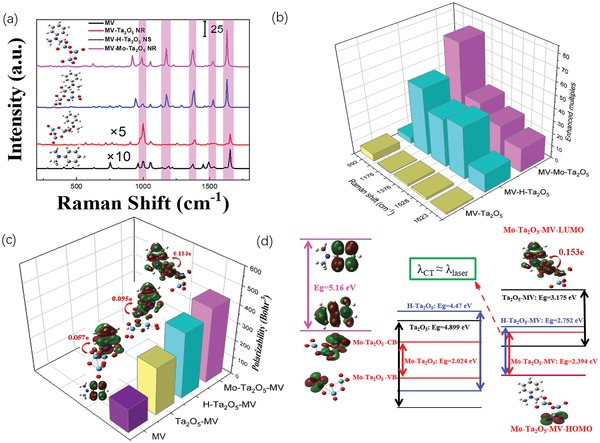
a) The calculated static Raman spectrum of MV and MV on different Ta_2_O_5_ SERS substrates (Ta_2_O_5_ NRs, Mo‐Ta_2_O_5_ NRs, and H‐Ta_2_O_5_ NSs). b) Enhanced multiples of calculated Raman modes of MV at 992, 1176, 1376, 1528, and 1632 cm^−1^on different Ta_2_O_5_ substrates. c) Calculated polarizabilities of the single MV, the MV adsorbed on Ta_2_O_5_ NR, H‐Ta_2_O_5_, and the Mo‐Ta_2_O_5_, respectively. d) The molecular orbital illustrations of the MV, different Ta_2_O_5_ clusters and MV‐Ta_2_O_5_ complexes.

The partial density of states (PDOS) and band structure diagrams of the Ta_2_O_5_ NR, Mo‐Ta_2_O_5_ NR, and H‐Ta_2_O_5_ NS substrates were calculated and are shown in Figure S5 (Supporting Information). Consistent with the experimental results, the calculated bandgap of the Mo‐Ta_2_O_5_ NRs became narrower (0.67 eV) than that of the Ta_2_O_5_ NRs (2.062 eV), which was due to the appearance of a tailed state energy level under the CB of Ta_2_O_5_ after replacing the Ta atom with Mo to form the Mo—O—Ta chemical bond. In contrast, both the CB and the VB positions shifted down due to the lower electron energy after hydrogenation and accordingly produced a narrower bandgap in H‐Ta_2_O_5_ (1.203 eV). Figure S6 (Supporting Information) compares the calculated and experimental Raman spectra of MV. The lines in the density functional theory (DFT)‐calculated Raman spectra of MV agree with the experimental results well, suggesting that the theoretical calculation was reasonable and feasible. The static Raman scattering spectra of a single MV molecule and MV adsorbed on the Ta_2_O_5_, H‐Ta_2_O_5_, and Mo‐Ta_2_O_5_ clusters are shown in **Figure** 5a. Notably, the Raman intensity of the MV molecule was apparently enhanced when it was adsorbed on H‐Ta_2_O_5_ especially the modes at 1176 and 1376 cm^−1^. Predictably, a more prominent enhancement in the lines at 1176, 1376, 1528, and 1632 cm^−1^ on Mo‐Ta_2_O_5_ can also be observed in Figure 5b, among which the primary and secondary SERS peaks were mainly due to the intensified stretching vibration of the benzene ring and the bending of the methyl group in MV after the incorporation of Mo atoms into Ta_2_O_5_, suggesting a very strong CT process between the MV molecules and element‐doped Ta_2_O_5_ substrates. To illustrate the increased SERS activity of the H‐Ta_2_O_5_ and Mo‐Ta_2_O_5_ substrates, the polarizabilities of the MV‐Ta_2_O_5,_ MV‐H‐Ta_2_O_5_ and MV‐Mo‐Ta_2_O_5_ complexes (Figure 5c), as well as the CT quantity and molecular orbital rearrangement of the MV‐H‐Ta_2_O_5_ complex and the MV‐Mo‐Ta_2_O_5_ complex were calculated. According to the calculated energy level distribution of MV, the H‐Ta_2_O_5_ and Mo‐Ta_2_O_5_ clusters and their complexes, a distinct electronic rearrangement was observed in these composite substrates, which adjusted the band structure of the systems and helped them make full use of the 532 nm excitation laser to excite the Raman signal. Relative to that of the probe molecule MV, the bandgaps of the MV‐H‐Ta_2_O_5_ and MV‐Mo‐Ta_2_O_5_ complexes were distinctly reduced to 2.75 and 2.39 eV, respectively, both of which were narrower than the MV‐Ta_2_O_5_ system (3.17 eV). The resonance excitation wavelengths for the CT process in these three MV‐Ta_2_O_5_, MV‐H‐Ta_2_O_5_ and MV‐Mo‐Ta_2_O_5_ complexes were 390, 451, and 519 nm respectively, among which the energy band and the CT resonance frequency of the MV‐Mo‐Ta_2_O_5_ complex (λ_CT_) best matched with the incident 532 nm excitation wavelength (λ_laser_) as follows

(4) $$\lambda_{\mathrm{CT}}\approx \lambda_{\mathrm{laser}}$$

and thus corresponded to the strongest SERS intensity from the CT resonance. Moreover, the redistributions of the charge density showed that the Mo—C bond served as the CT channel and promoted the CT process from the CB of Mo‐Ta_2_O_5_ to the highest occupied molecular orbital (HOMO) of MV with a CT amount of 0.153 e (Figure 5d). The amount of CT in H‐Ta_2_O_5_‐MV and Ta_2_O_5_‐MV were 0.095 and 0.057 e, respectively, both of which were inferior to that of Mo‐Ta_2_O_5_ (Figure 5c)_._ Simultaneously, the calculated polarizabilities of MV and MV adsorbed on the Ta_2_O_5_ NR, H‐Ta_2_O_5_, and Mo‐Ta_2_O_5_ substrate clusters were 139, 273, 286, and 425 Bohr^3^, respectively (Figure 5c). The CT resonance frequency, the amount of CT and the calculated polarizability results all suggested sequentially stronger CT and Raman signal from the Ta_2_O_5_ NRs and H‐Ta_2_O_5_ NSs to the Mo‐Ta_2_O_5_ NRs, and all of the above calculation results were consistent with the experimental results. By regulating the energy band of Ta_2_O_5_ NRs via elemental doping, we fulfilled the quasi‐approximation λ_mol_ ≈ λ_CT_ ≈ λ_laser_, and thus both the PICT resonance and molecular resonance synergistically contributed to the Raman intensity of the 15%‐Mo‐Ta_2_O_5_ substrates.

In addition to the MV molecular resonance and the PICT resonance between MV molecules and Ta_2_O_5_ NRs from the CM, the contribution of the EM enhancement around the “gap” and “tip” of anisotropic Ta_2_O_5_ NRs to the surface‐enhanced Raman intensity under 532 nm laser excitation should not be underestimated. The finite element method (FEM) was used to simulate the surface electric field distribution of one Ta_2_O_5_ NR, two Ta_2_O_5_ NRs with a gap position and Ta_2_O_5_ NS. The strongest electric field enhancement factor for the single Ta_2_O_5_ NR under a 532 nm excitation laser was only 3 (Figure S7a‐1, Supporting Information). Usually, there are many Ta_2_O_5_ NRs stacked and crossed together randomly in our sample, and many crossing and gap locations appear. Certainly, the strongest electric field enhancement factor can even reach ≈9 at the “angular” and “gap” positions of the two Ta_2_O_5_ NRs under irradiation with a 532 nm laser (Figure S7b‐1, Supporting Information), which can be assumed to be modest “hot spots,” which are well known in plasmonics. Thus, the electromagnetic resonance can achieve three orders of magnitude of enhancement in SERS when the Ta_2_O_5_ NRs are in a good relative position. Considering the high density of hot spots within the powder of aggregated Ta_2_O_5_ NRs, the EM contribution to the SERS enhancement can be even higher. Thus, the strong electric field enhancement at the angular and gap positions of the Ta_2_O_5_ NRs can well explain the strong electromagnetic enhancement of our Ta_2_O_5_ NRs substrate under 532 nm excitation light. For the H‐Ta_2_O_5_ NS substrates, the largest simulated electric field enhancement factor under the incident 532 nm laser was 3.6 (Figure S7c‐1, Supporting Information). Although there was a stronger electric field enhancement by the Ta_2_O_5_ NS than by the single Ta_2_O_5_ NR, the smaller size and sharper corners of the Ta_2_O_5_ NRs made them easier to generate stronger electric field enhancement when they were aggregated, thus demonstrating a stronger electric field distribution around the “gap” and “tip” of anisotropic Ta_2_O_5_ NRs and the possible greater contribution of the NRs (6 nm in diameter) than the NSs (250 nm in diameter) to the surface‐enhanced Raman intensity. We further simulated the electric field distribution around these Ta_2_O_5_ substrates at two different excitation wavelengths (633 and 785 nm), both of which induced a slightly weaker electric field than the 532 nm laser in all three models and verified that 532 nm excitation light provided the best electromagnetic field enhancement. Hence, among the current excitation lasers, the 532 nm laser was the most compatible with the electromagnetic resonance of our Ta_2_O_5_ substrate; here, the λ_EM_ was considered to be approximately equal to λ_laser_ as follows

(5) $$\lambda_{\mathrm{EM}}\approx \lambda_{\mathrm{laser}}$$

However, the 15%‐Mo‐Ta_2_O_5_ substrates had poor performance when detecting 10^−6^ m MV under irradiation with 633 and 785 nm lasers (Figure S9c, Supporting Information), which was most likely caused by the mismatch between the excitation laser and the molecular and CT frequencies.

In summary, based on our presented “coupled resonance” strategy, extraordinary SERS performance of our 15%‐Mo‐Ta_2_O_5_ NRs for the carefully selected dye MV was obtained, and can be attributed to the synergistic effect of the molecular resonance (λ_mol_ ≈ λ_laser_), PICT resonance (λ_CT_ ≈ λ_laser_), and electromagnetic resonance (λ_EM_ ≈ λ_laser_), which was realized by regulating the PICT and electromagnetic resonance frequencies of our Ta_2_O_5_ substrates to be quasi‐equivalent to the given 532 nm laser by energy engineering through element doping. This result can be well understood and described by a recent theoretical model of SERS,^47^ as various mesoscopic and microscopic strategies of SERS have been identified and discussed. The mechanisms of molecular resonance (λ_mol_ ≈ λ_laser_) and PICT (λ_CT_ ≈ λ_laser_) belong to the set of microscopic strategies, which can be described by enhanced molecular Raman cross‐section or polarizability, whereas the mechanism of electromagnetic resonance (λ_EM_ ≈ λ_laser_) belongs to the category of mesoscopic strategies, which are closely related to the well‐established local field enhancement of Raman scattering. Importantly, the “coupled resonance” strategy is expected to be further applied as a guide to design different semiconductor SERS substrates for a better detection of many other molecules in the future.

### 2.4 The Coexistence of Photocatalytic Degradation Ability and its Influence on SERS Detection

**Figure 6:**
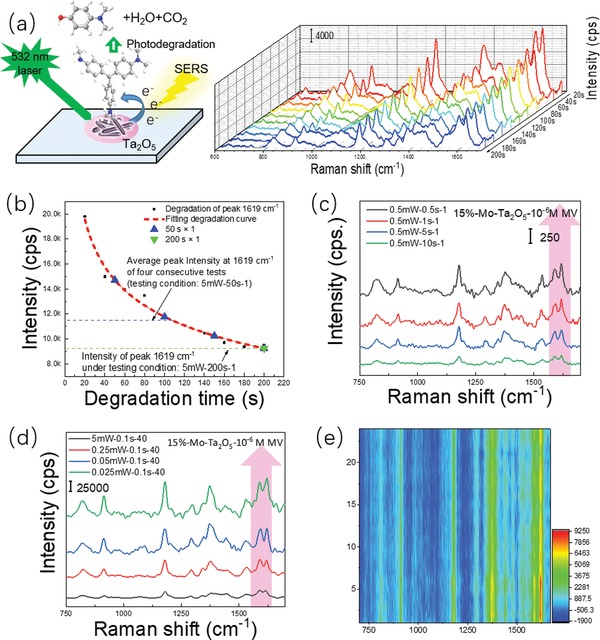
a) SERS spectra of 10^−6^ m MV degraded on 15%‐Mo‐Ta_2_O_5_ substrate under the 532 laser. b) Simulated dynamics curve of 532 nm‐laser‐degradated 10^−6^ m MV on 15%‐Mo‐Ta_2_O_5_ substrate. c) Comparison of detected SERS signal of 10^−6^ m MV on 15%‐Mo‐Ta_2_O_5_ substrate with different single detected time (0.5, 1, 5, and 10 s) after homogenization under the fixed laser power (0.5 mW). d) Comparison of detected SERS signal of 10^−6^ m MV on 15%‐Mo‐Ta_2_O_5_ substrate with different laser powers after homogenization, the 532 nm incident laser with varying power (5, 0.25, 0.05, and 0.025 mW) were used to detect the MV SERS signal when fixing the same single time and the accumulation times (0.1 s × 40). e) 2D‐Mapping of the SERS signal of 10^−7^ m MV on the surface of the 15%‐Mo‐Ta_2_O_5_ substrate.

Whether there were factors other than the disparity of the dominant enhancement mechanism that constrained the SERS performance improvement of semiconductors was an interesting research focus. An unusual phenomenon was observed in our SERS experiment. The collected SERS signal decay took place when we repeatedly detected MV at a fixed point of Ta_2_O_5_ substrates (**Figure** 6a). It was suggested that concomitant photoinduced degradation of probed molecules in the SERS detection process would lead to the SERS signal decay on the photocatalytic semiconductor SERS substrate. There seems to be a contradiction in our semiconductor SERS substrate. The photodegradation degree of probed molecules was also increased when a stronger SERS signal was detected with a higher laser power. In addition to the inherent photobleaching phenomenon of dye molecules by a strong laser, the semiconductor's unique photocatalytic degradation of dye molecules can further intensify the SERS signal decay of dye molecules on the photocatalytic semiconductor SERS substrate. This effect may be an important reason why many semiconductor SERS substrates are less sensitive than precious metals, and why their detection limit is difficult to break through. Ultrafast investigations using MV‐Ta_2_O_5_, pure Ta_2_O_5_, and pure MV samples revealed the inevitable impact of photoinduced degradation on the carrier behavior in MV‐Ta_2_O_5_, which is within the time scale of the Raman test (see experimental details in the Supporting Information 4). As shown in Figure S11 (Supporting Information), a clear distinction between the dynamics of MV‐Ta_2_O_5_ and pure Ta_2_O_5_ can be observed. As illustrated in Figure S12 (Supporting Information), laser light illumination can also induce direct photobleaching of the MV sample without Ta_2_O_5_. Therefore, a) the photocatalytic degradation of MV molecules by Ta_2_O_5_ and b) the direct photobleaching for the MV molecules (without Ta_2_O_5_) together contribute to the photoinduced decay. Each of them has a higher decay rate with increasing laser power. Because the photoinduced carrier behavior is also related to the PICT process, the photocatalytic degradation process of the MV molecules under the catalysis of the Ta_2_O_5_ substrates should not be ignored during Raman measurements. Moreover, we examined the photocatalytic degradation ability of our Ta_2_O_5_ substrate for MV molecules under irradiation with simulated daylight (Figure S8, Supporting Information). Despite the poor photocatalytic degradation ability of Ta_2_O_5_ NRs, the self‐cleaning ability of the 15%‐Mo‐Ta_2_O_5_ substrate under simulated visible light was obvious enhanced (quickly degrading 82% of the MV molecules within 10 min), demonstrating that the 15%‐Mo‐Ta_2_O_5_ substrate possessed both optimal photocatalytic degradability and superior SERS performance. More precisely, another experiment was performed to determine whether the photodegradability of the semiconductor had an effect on our Raman test results. We focused the 532 nm incident laser at a point on the sample and collected the SERS signal every 20 s. It can be observed from Figure 6a that significant SERS signal attenuation appeared during the maintenance of single‐point detection. Simultaneously, the simulated dynamics curve of 532 nm‐laser‐degraded MV on the 15%‐Mo‐Ta_2_O_5_ substrate in Figure 6b indicated that shortening the single‐point detection time in SERS measurements were beneficial to reduce the impact of photoinduced degradation on SERS detection. At the same time, laser power was also a significant factor during the photoinduced degradation of MV. Hence, favorable measurement parameters were very important and should be critically selected. As the time for collecting data from a single point was shortened from 10 to 0.5 s, the detected SERS signals of MV (after normalization) on the same 15%‐Mo‐Ta_2_O_5_ substrate were obviously enhanced (Figure 6c), which fully matched our expectation because the detected SERS signal would be much closer to the unattenuated true SERS signal with the sharp shortening of the single‐point detection time. At the same time, the SERS signal decay of MV molecules was further intensified by the laser with higher power, as shown in Figure 6d (normalized from Figure S9a (Supporting Information) to eliminate the divergence from different powers). Even the SERS signal of MV could not be detected on the 15%‐Mo‐Ta_2_O_5_ substrate due to the severe degradation of MV when the incident laser power was large enough (50 mW) (Figure S9b, Supporting Information), which provided the most convincing evidence for our previous conjecture. In the presence of photodegradation, the molecular Raman scattering cross‐section or polarizability could be significantly decreased, perhaps because of molecular chemical structure changes or even damage under strong laser irradiation.^47^ Therefore, we expect that rapidly measuring the SERS signal of those degradable probe molecules on the surface of photocatalytic semiconductor SERS substrates via ultrafast Raman spectroscopy is probably helpful for discovering more sensitive semiconductor SERS‐active materials.

Undoubtedly, the single‐point‐detected SERS signal was not reproducible for the photocatalytic Ta_2_O_5_ SERS substrates because our substrates were photodegrading the probed MV molecules during the SERS measurements. Notably, our sample can still output stable SERS signals as long as the detected position was slightly changed (Figure 6e), because each incident spot was only 1 µm in size and much smaller than our substrates with a diameter over 1 cm. Based on the SERS signal intensity from the 2D‐mapping of 10^−7^ m MV on the 15%‐Mo‐Ta_2_O_5_ substrate, the relative standard deviation (RSD) of the Raman lines at 1179 and 1617 cm^−1^ were calculated as 12.5% and 13.5%, respectively (Figure S10a,b, Supporting Information). Thus, a good SERS signal reproducibility can be achieved in our 15%‐Mo‐Ta_2_O_5_ substrates. Moreover, our 15%‐Mo‐Ta_2_O_5_ substrates also have a good stability to storage in air atmosphere without the problem of easy agglomeration and oxidation failure like noble metal nanoparticles, it can still show good SERS performance after storage in air for more than three months (Figure S10c, Supporting Information).

## 3 Discussion

In this work, the important “coupled resonance” strategy of matching SERS selection rules was presented to optimize the SERS performance of a novel semiconductor SERS‐active substrate Ta_2_O_5_ by energy band engineering, which was implemented through multiple elemental doping methods (hydrogen‐reduced H‐Ta_2_O_5_ NSs and high‐valence Mo‐doped Ta_2_O_5_ NRs). Among them, the Mo‐doped Ta_2_O_5_ substrate exhibited a remarkable SERS sensitivity with an EF of 2.2 × 10^7^ and a very low detection limit of 9 × 10^−9^ m for the carefully selected MV molecules, which, to the best of our knowledge, is one of the highest sensitivities among those reported for semiconductor SERS substrates under a specific laser excitation wavelength of 532 nm and even parallels that of a noble metal without “hot spots.” This extraordinary SERS performance can be attributed to the synergistic realization of molecular, PICT and electromagnetic resonance on the Mo‐doped Ta_2_O_5_ NR substrates under the 532 nm excitation laser according to our quasi‐approximate equation, which may provide guidance for the design of highly sensitive semiconductor SERS substrate materials in the future. Simultaneously, unambiguous and non‐neglectable photodegradation of MV on the Ta_2_O_5_ substrate within the time scale of the Raman test was also revealed by ultrafast spectroscopy, and thus we proposed an important point of view for the first time: the photocatalytic ability of many conventional semiconductor SERS substrates, such as ZnO, TiO_2_, and CdS, may be an important factor that limits their SERS performance. Finally, we expect that rapid measurements of the SERS signal of those degradable probe molecules on the surface of photocatalytic semiconductor SERS substrates via ultrafast Raman spectroscopy is probably helpful for discovering more sensitive semiconductor SERS‐active materials.

## 4 Experimental Section

*Synthesis of Ta_2_O_5_ NRs, H‐Ta_2_O_5_, and Mo‐doped Ta_2_O_5_ Substrates*: The Ta_2_O_5_ NRs were synthesized by the hydrothermal method according to the literature.^27^ Analytical grade hydrofluoric acid (HF) was purchased from Aladdin Co., Ltd. and used to dissolve 2.0 g of Ta_2_O_5_ powder (analytically pure, Aladdin), which was reprecipitated from the mixture by adjusting the pH to 9. Then, the precursor was obtained by redissolving the freshly formed precipitate into a 60 mL mixed solution containing hydrogen peroxide (analytically pure, 30 wt% in H_2_O) and ammonia (analytically pure, Sinopharm Chemical Reagent Co., Ltd) at a volume ratio of 5:1. Before the hydrothermal reaction, the precursor was slowly heated at 60 °C for 1 h to promote the decomposition of H_2_O_2_, and then it was transferred into a PPL‐lined stainless‐steel autoclave (100 mL) to carry out the reaction at 240 °C for 36 h. Finally, the resultant product was thoroughly rinsed with deionized water and ethanol several times and dried naturally for subsequent characterization. The synthetic process for Mo‐doped Ta_2_O_5_ was fundamentally similar to the above process. The Mo‐doped Ta_2_O_5_ samples were prepared by mixing a certain amount of ammonium molybdate (analytically pure, Sinopharm Chemical Reagent Co., Ltd.) into the precursor. The molar ratios of the Mo added to Ta were 10%, 15%, and 20%, and the resulting samples were labeled 10%‐Mo‐Ta_2_O_5_, 15%‐Mo‐Ta_2_O_5_, and 20%‐Mo‐Ta_2_O_5_, respectively. The H‐Ta_2_O_5_ sample was prepared by hydrogenating Ta_2_O_5_ NRs in a H_2_/N_2_ atmosphere (volume ratio: 1/9) at 1200 °C for 6 h.

*Characterization of Ta_2_O_5_ NRs, H‐Ta_2_O_5_, and Mo‐doped Ta_2_O_5_ Substrates*: The micro‐morphology of Ta_2_O_5_ NRs, Mo‐doped Ta_2_O_5_ NRs, and H‐Ta_2_O_5_ NSs was determined by a JEM‐2100F field‐emission source TEM, and HRTEM was used to further obtain the size and inter‐planar spacing of the lattice fringes of these different Ta_2_O_5_ substrates. The surface element and chemical state were examined via XPS with a Thermo Fisher Scientific ESCAlab250. Characterization of the crystallinity was performed by XRD. XAS was carried out at the photoemission endstation at the BL10B beamline in the National Synchrotron Radiation Laboratory (NSRL). All XAS spectra were recorded in total electron yield (TEY) mode using a sample current measurement. UV–vis diffuse reflectance spectra were used to obtain the information on the optical properties and energy band structure of the substrates. All the Raman and SERS spectra were collected on a Renishaw inVia Reflex Raman spectrometer with excitation wavelengths of 532, 633, and 785 nm. For all Raman tests under each changed condition, a fresh position on the same sample was re‐selected.

*SERS Activity Measurement of Substrates*: An MV aqueous solution with a concentration of 10^−6^–9 × 10^−9^ m were used for the SERS detection. Raman spectra were then collected by dropping a dose of MV aqueous solution (10^−6^–9 × 10^−9^ m) with a volume of 1 µL on the surface of the samples and then drying for 1 h. The excitation wavelength was 532 nm and a 10 ×/0.30 objective lens was used to focus the laser beam. SERS activity measurement of substrates were conducted under a laser power of 5 mW and a total exposure time of 20 s, which were acquired with a single time of 1 s and accumulation times of 20 (1 s × 20). The signal intensity of each Raman data was averaged from three detected Raman signals from three randomly selected points on the surface of the samples. The 2D‐Mapping SERS signals of 10^−7^ m MV on the surface of 15%‐Mo‐Ta_2_O_5_ substrate were collected under the laser power of 5 mW and a total exposure time of 8 s.

*Photodegradation of MV on Ta_2_O_5_ Substrates under Simulated Sunlight*: First, 0.003 g of Ta_2_O_5_ sample (Ta_2_O_5_ NR, 15%‐Mo‐Ta_2_O_5_, and H‐Ta_2_O_5_) were first immersed into 3 mL of 10^−5^ m MV aqueous solution. Then, the 10^−5^ m MV aqueous solution containing the Ta_2_O_5_ NR, 15%‐Mo‐Ta_2_O_5_, and H‐Ta_2_O_5_ sample at the bottom of the cuvette was directly used in order to clearly see the variety of the absorption peak of MV. These samples were irradiated with simulated daylight using a xenon lamp source, and the UV–vis spectra of the MV photocatalyzed by the Ta_2_O_5_ substrates at different irradiation times were recorded and compared.

*Photodegradation of MV on Ta_2_O_5_ Substrates under the 532 nm Laser*: The SERS spectra of the MV molecules on the 15%‐Mo‐Ta_2_O_5_ substrate were collected at one fixed irradiation point with an excitation wavelength of 532 nm at a laser power of 0.5 mW and a total exposure time of 20 s (1 s × 20). The next nine complete SERS signals were continually collected in a constant position according to this condition to acquire the SERS signal of 532 nm‐laser‐degraded MV on the 15%‐Mo‐Ta_2_O_5_ substrate. Under the fixed laser wavelength and laser power (532 nm and 0.5 mW), the detected SERS signal (after homogenization) of 10^−6^ m MV on 15%‐Mo‐Ta_2_O_5_ substrate at different detected time (0.5, 1, 5, and 10 s) were compared to study the effect of photocatalysis on SERS performance under the different detection time. When fixing the same detected time and the accumulation times (0.1 s × 40) under the 532 nm incident laser, the detected SERS signal (after homogenization) of 10^−6^ m MV on 15%‐Mo‐Ta_2_O_5_ substrate with varying laser powers (5, 0.25, 0.05, and 0.025 mW) were compared to study the effect of photocatalysis on SERS performance under the different laser powers.

## Conflict of Interest

The authors declare no conflict of interest.

## Supporting information

Supplementary
