# Supplementary material for: A Novel Ultra‐Sensitive Semiconductor SERS Substrate Boosted by the Coupled Resonance Effect
Source: Adv Sci (Weinh). 2019 Apr 16;6(12):1900310. doi: 10.1002/advs.201900310 (PMC6662085; doi:10.1002/advs.201900310)
Supplement: Supplementary file 1 — Supplementary [file ADVS-6-1900310-s001.pdf]

Copyright WILEY-VCH Verlag GmbH & Co. KGaA, 69469 Weinheim, Germany, 2019.

ADVANCED SCIENCE Open Access

Supporting Information

for *Adv. Sci.,* DOI: 10.1002/advs.201900310

A Novel Ultra-Sensitive Semiconductor SERS Substrate Boosted by the Coupled Resonance Effect

Lili Yang, Yusi Peng, Yong Yang, Jianjun Liu, Haoliang Huang, Bohan Yu, Jimin Zhao, Yalin Lu, Zhengren Huang, Zhiyuan Li, and John R. Lombardi*

ASSOCIATED CONTENT

Supporting Information

A Novel Ultra-Sensitive Semiconductor SERS Substrate Boosted by the Coupled Resonance Effect

Lili Yang, [1,2,3] *Yusi Peng,* [1,2,3] *Yong Yang,* [1,2,3]* *Jianjun Liu,* [1] *Haoliang Huang,* [4] *Bohan Yu,* [2,5] *Jimin Zhao,* [2,5] *Yalin Lu,* [4] *Zhengren Huang,* [1] *Zhiyuan Li* [6] *and John R. Lombardi* [7]

[1] State Key Laboratory of High Performance Ceramics and Superfine Microstructures, Shanghai Institute of Ceramics, Chinese Academy of Sciences, 1295 Dingxi Road, Shanghai 200050, People's Republic of China.

[2] University of Chinese Academy of Sciences, No.19(A) Yuquan Road, Beijing 100049, People's Republic of China.

[3] Center of Materials Science and Optoelectronics Engineering, University of Chinese Academy of Sciences, Beijing 100049, People's Republic of China.

[4] National Synchrotron Radiation Laboratory, University of Science and Technology of China, Hefei 230026, People's Republic of China.

[5] Beijing National Laboratory for Condensed Matter Physics and Institute of Physics, Chinese Academy of Sciences, Beijing 100190, People's Republic of China.

[6] South China University of Technology, Guangzhou 510640, Guangdong, People's Republic of China.

[7] Department of Chemistry, The City College of New York, 160 Convent Avenue, New York, NY 10031, USA.

*Corresponding Author: E-mail: yangyong@mail.sic.ac.cn

Supporting Information 1

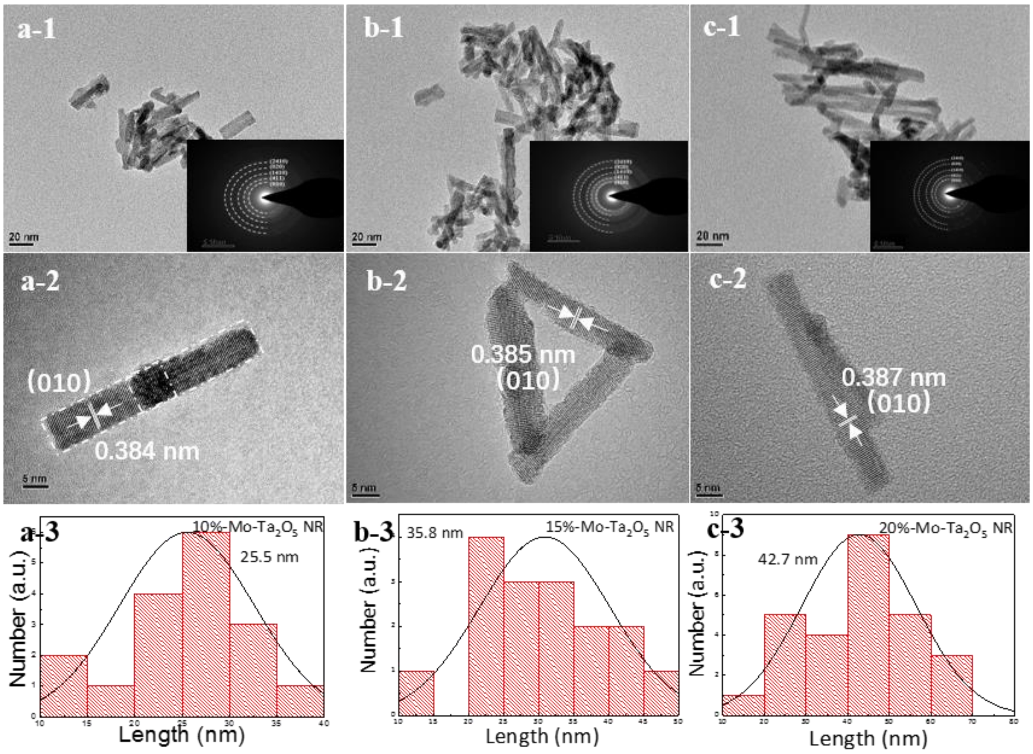

Figure S1. TEM (1), HRTEM (2) and Length distribution histogram (3) images of (a) 10%-Mo-Ta_2O_5 NR, (b) 15%-Mo-Ta_2O_5 NR, and (c) 20%-Mo-Ta_2O_5 NR substrates. Inset in Figure S1a-1: SAED pattern of 10%-Mo-Ta_2O_5 NR; Inset in Figure S1b-1: SAED pattern of 15%-Mo-Ta_2O_5 NR; Inset in Figure S1c-1: SAED pattern of 20%-Mo-Ta_2O_5 NR.

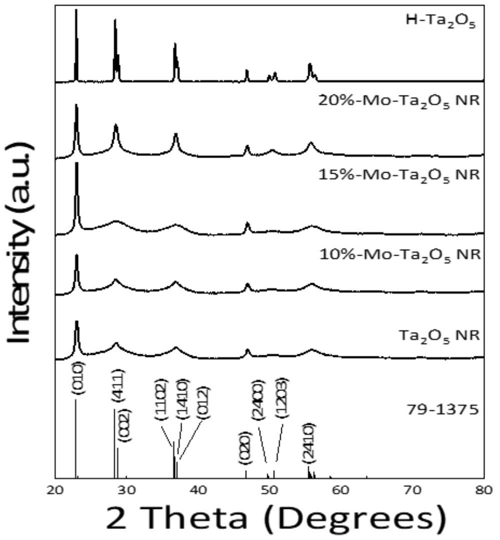

Figure S2. XRD patterns of different Ta_2O_5 substrates.

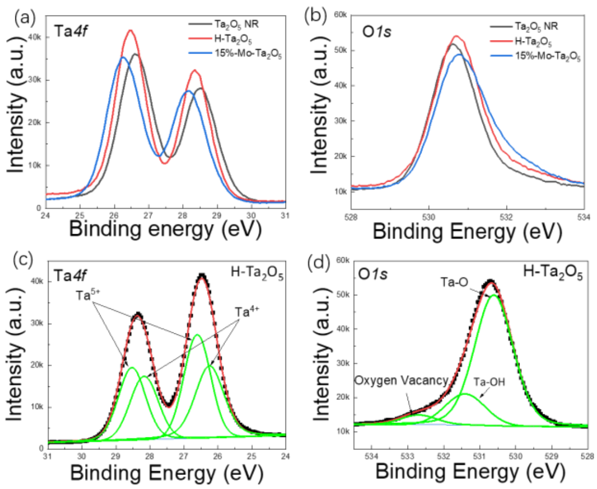

Figure S3. (a) Ta*4f* and (b) O*1s* XPS spectrum in different Ta_2O_5 substrates (Ta_2O_5 NRs, H-Ta_2O_5 NSs and 15%-Mo-Ta_2O_5 NRs); (c) Ta*4f* XPS spectrum of H-Ta_2O_5 NSs substrates and (d) O*1s* XPS spectrum of H-Ta_2O_5 NSs substrates.

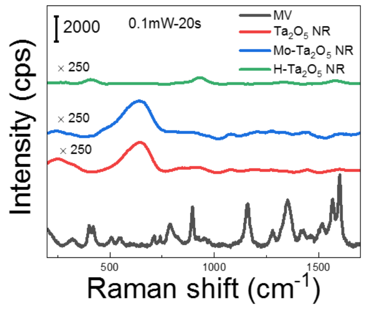

Figure S4. Raman spectroscopy of MV power and Ta_2O_5 substrates.

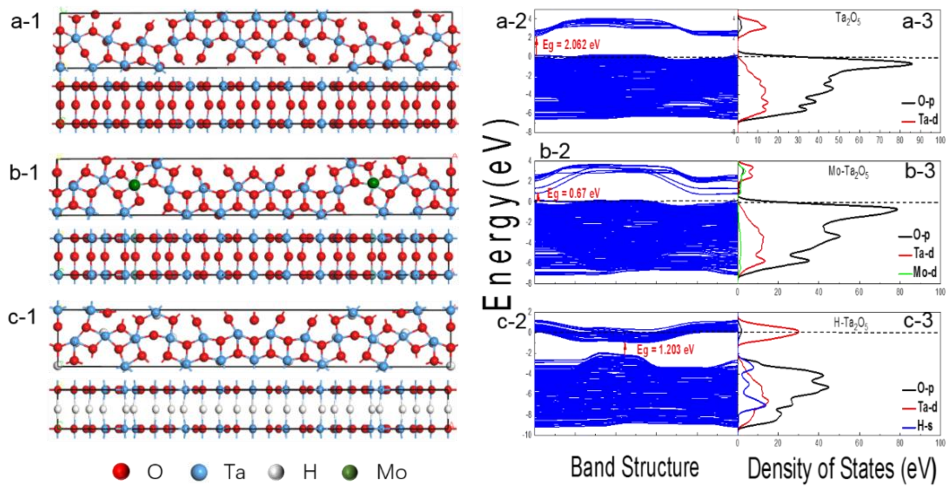

Figure S5. The crystal structure of (a-1) Ta_2O_5, (b-1) Mo-Ta_2O_5 and (c-1) H-Ta_2O_5 substrates; Band structure of (a-2) Ta_2O_5, (b-2) Mo-Ta_2O_5 and (c-2) H-Ta_2O_5 substrates; Partial density of states of (a-3) Ta_2O_5, (b-3) Mo-Ta_2O_5 and (c-3) H-Ta_2O_5 substrates.

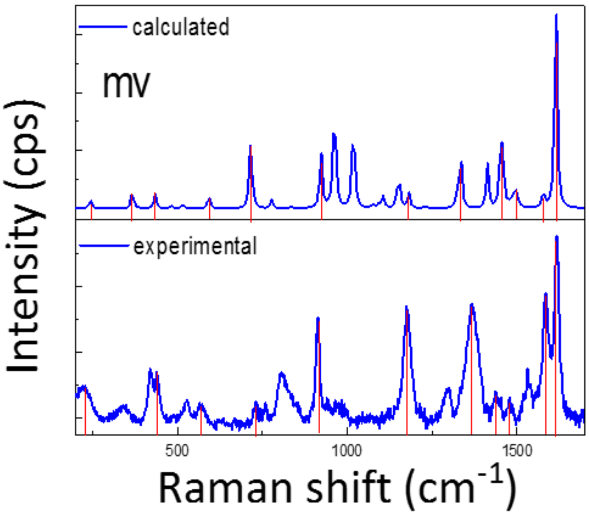

Figure S6. Comparison of calculated and experimental Raman spectrum of MV.

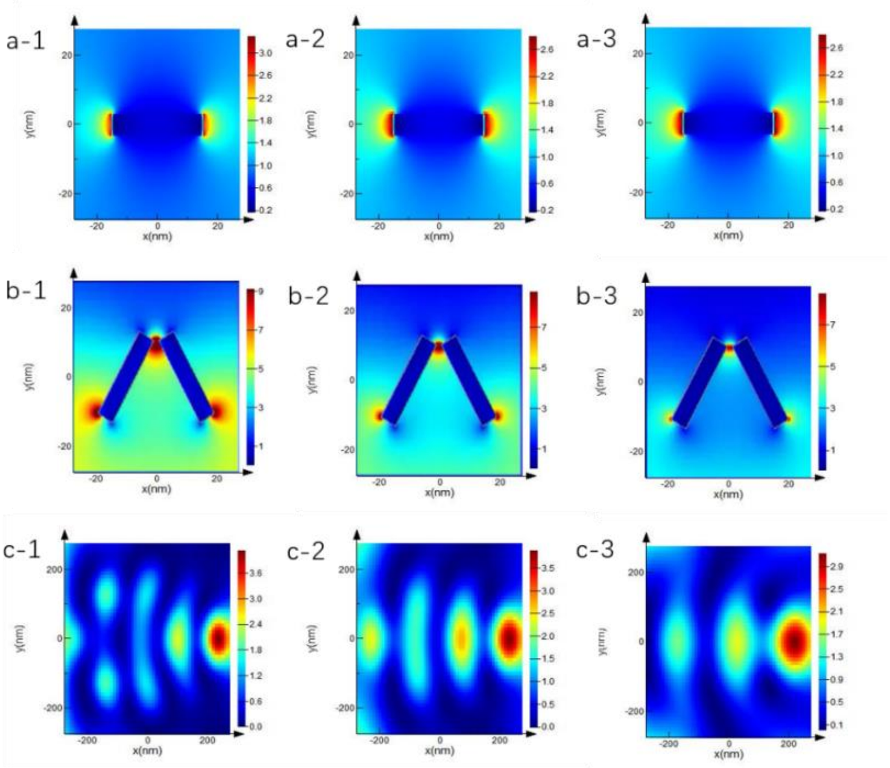

Figure S7. Calculated electric field distributions for (a) one Ta_2O_5 NRs with 6 nm diameter and 30 nm length; (b) two Ta_2O_5 NRs with an angle of 60 degrees and (c) Ta_2O_5 NSs with 250 nm diameter and 3nm thickness under the excitation laser of (1) 532 nm, (2) 633 nm and (3) 785 nm.

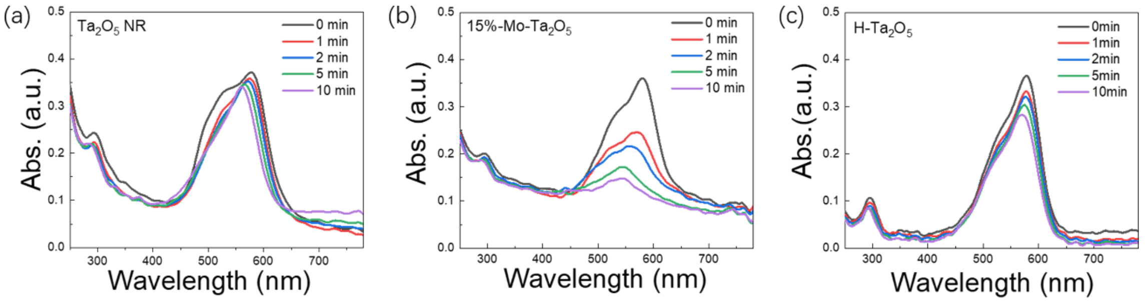

Figure S8. Simulated daylight-catalytic degradation of 10^{-5} M MV on (a) Ta_2O_5 NRs, (b) 15%-Mo-Ta_2O_5 NRs and (c) H-Ta_2O_5 NSs substrates.

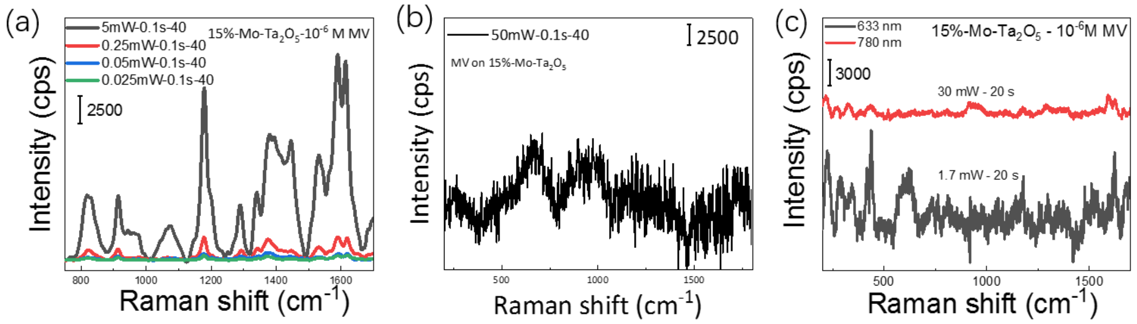

Figure S9. (a) Detected SERS signal of 10^{-6} M MV on 15%-Mo-Ta_2O_5 substrate with different laser power, the 532 nm incident laser with varing power (5 mW, 0.25 mW, 0.05 mW and 0.025 mW) were used to detect the MV SERS signal when fixing the same single time and the accumulation times (0.1 s × 40); (b) Detected SERS signal of 10^{-6} M MV on 15%-Mo-Ta_2O_5 substrate under the laser power of 50 mW and a severe degradation of MV; (c) Detected SERS signal of 10^{-6} M MV on 15%-Mo-Ta_2O_5 substrate under 633 nm (black line) and 785 nm laser (red line), the detected power were 1.7 mW and 30 mW respectively.

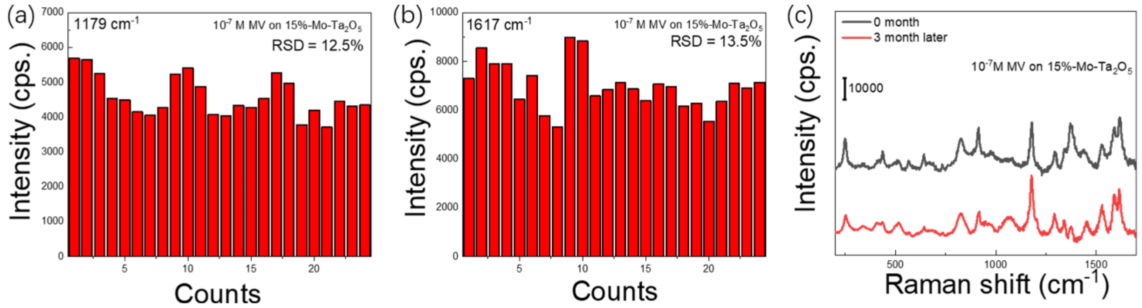

Figure S10. The RSD of the intensities at the Raman lines 1179 cm^{-1} (a) and 1617 cm^{-1} (b) of MV, which were acquired from the 2D-Mapping of SERS signal of 10^{-7} M MV on the surface of 15%-Mo-Ta_2O_5 substrate. (c) Detected SERS signal of 10^{-7} M MV on 15%-Mo-Ta_2O_5 substrate before and after 3 months with the laser power of 5 mW and a total exposure time of 20 s (1 s × 20).

Table S1. Data of peak a and peak d in the O K-edge XAS spectra of the different Ta_2O_5 samples.

	Ta_2O_5 NR	H-Ta_2O_5	10%-Mo-Ta_2O_5	15%-Mo-Ta_2O_5	20%-Mo-Ta_2O_5
**Area ratio of Peak a (%)**	5.235	5.852	4.719	5.996	4.854
**FWHM of Peak d (eV)**	6.519	6.217	8.235	5.138	6.079

Table S2. Raman shifts of SERS characteristic peaks and corresponding assignments and EFs.

**Raman shift (cm^{-1})**		**EF**	**Assignments** **ν, stretching (s, symmetric; as, asymmetric); δ, bending**
MV (soild)	MV (on 15%-Mo-Ta_2O_5)		
1620	1617	2.2×10^7	
1584	1587	1.6×10^7	
1535	1529		$\nu(C_{ring}N)/\delta(CCC)_{ring}$
1443	1435		$\delta_{as}(CH_3)$
1371	1371	4.1×10^6	$\nu_{as}(CC_{center}C)/\delta(CCC)_{ring}/\delta(CH)$
1299	1295		$\nu_{as}(CC_{center}C)/\delta(CCC)_{ring}/\delta(CH)$
1177	1179	1.6×10^7	$\nu_{as}(CC_{center}C)$
974	973		
914	915	8.8×10^6	$\delta(CC_{center}C)$
808	826		
442	437		$\delta(CNC)$

Table S3. Reported enhancement factors (EFs) on different semiconductor nanostructure substrates.

Supporting Information 2

Enhancement factor (EF) calculation

The EF of the 15%-Mo-Ta_2O_5 substrate as the best SERS active substrate was calculated according to the general formula[11]:

$$EF=\frac{I_{SERS}}{I_{bulk}}\times\frac{N_{bulk}}{N_{SERS}} \quad (1)$$

$$N_{bulk}=\frac{\rho h}{M}A_{spot}N_{A} \quad (2)$$

$$N_{SERS}=CVN_{A}\frac{A_{spot}}{A_{substrate}} \quad (3)$$

In formula (1), I_{Raman} and I_{SERS} were respectively the integral intensity of the selected Raman peak during the Raman and SERS measurement. N_{bulk} and N_{SERS} were respectively the

Substrates	Probes	EF/Detection Limits	Excited wavelength (nm)
**Porous ZnO nanosheets[1]**	4-MBA	$10^3/10^{-6}$M	514.5
**sea urchin-like $W_{18}O_{49}$[2]**	R6G	3.4±0.41 × $10^5/10^{-7}$M	532.8
**TiO_2 photonic microarray[3]**	MB	2× 10^4 /6 × 10^{-6} M	532
**Amorphous ZnO nanocages[4]**	4-Mpy	6.62 × 10^5	633
**Oxygen-incorporated MoS_2[5]**	R6G	1.4 × 10^5 /10^{-7} M	532.8
**Nb_2O_5 nanoparticles[6]**	MeB	7.1 × 10^7 /10^{-6} M	780
**Amorphous MoO_3[7]**	R6G	1.8 × 10^7 /10^{-8} M	532
**Amorphous Rh_3S_6 microbowls[8]**	R6G	10^5 /10^{-7} M	647
**MoO_{3-x} quantum dots[9]**	R6G	10^6 /10^{-9} M	532
**Single Cu_2O superstructure particles[10]**	R6G	8 × 10^5 /6 × 10^{-9} M	647
**Ta_2O_5 NRs（This work）**	MV	1.5 × 10^4 / 10^{-6} M	532
**H-Ta_2O_5 NSs（This work）**	MV	3.2 × 10^6 / 10^{-7} M	532
**Mo-doping Ta_2O_5 NRs（This work）**	MV	2.2 × 10^7 /9 × 10^{-9} M	532

average number of MV molecules in the scattering volume for Raman and SERS detection. In formula (2), the average number of MV molecules in the scattering region for Raman detection was calculated with the molar mass (M = 408.03 g/mol) and the density (ρ = 1.109 g/cm^3) of bulk MV. The N_A was Avogadro constant, the A_{spot} was the irradiation area of the laser beam with a diameter of 2 μm and the confocal depth h was 21 μm.[12] In formula (3), a dosage of MV aqueous solution with the volume of V (1 μL) and the concentration of C (10^{-8} M) was dropped on the 15%-Mo-Ta_2O_5 substrate and spread to a circle of about 0.60 cm in diameter and an area of $A_{substrate}$. According, for SERS peak located at 1617 cm^{-1},

$$\frac{N_{bulk}}{N_{SERS}} = \frac{\rho h A_{substrate}}{CVM} = 8.07\times10^7.$$

Thus,

$$EF = 2.2 \times 10^7.$$

Supporting Information 3

In this paper, the first principle calculation method based on density functional theory[13] (DFT) was used to complete the geometric optimization and the electronic structural calculation of Ta_2O_5 crystal. The periodic boundary condition was used in the calculation process, the local density approximation[14] (LDA) method was applied for the inter-electronic exchange correlation energy, and the ultra-soft potential (Ultrasoft) was used to achieve the interaction potential between ion core and valence electrons. In the wave vector K-space, the cut-off energy of plane wave was chosen as 400 eV, the Brillouin zone (integral = 1 × 4 × 3) was summed according to the special K-point of Monkors-Park.[15] The special K points summed for the Brillouin area. When the total energy change of the system stable within

5×10^{-6} eV, the force acting on each atom in the unit cell, the residual stress of the unit cell and the tolerance deviation less than 0.01 eV/Å, 0.01 GPa and 1×10^{-4} Å respectively, the convergence state of the system was reached. We considered the weak interaction during the geometric optimization process. The added U values were respectively 3.0 and 4.38 for the Ta and Mo atom when considering the d orbital electrons. And then, the Gauss09 program was used to calculate the ground state geometric optimization, static Raman spectroscopy, charge distribution and molecular orbital properties of the Mo-doped and hydrogenated Ta_2O_5 systems with the mixed exchange functional B3LYP method. To ensure that all structures were in a stable state, we optimized the probe molecule and cluster models without virtual frequencies. The C, H, O and N atoms in the adsorption system were described by the 6-311+G (d, p) group including a polarization function and a diffusion function. The Lanl2dz basic group was selected for the transition metal Mo and Ta atoms. It has been verified that both the B3LYP/6-311+G(d,p) and the B3LYP/Lanl2dz basic group level were sufficient to describe the interaction between organic molecules and transition metal compound clusters, and all calculations were performed under the default convergence criteria of Gauss09.[16] Based on the DFT, the influence of elemental Mo doping and H incorporation on the electronic structure of Ta_2O_5 was studied by the first principle. We constructed a crystal structure of Ta_2O_5 NRs with a space group of Pmm2 (Figure S5a-1), and then replaced a Ta atom with a larger Mo atom to obtain a crystal model of Mo-Ta_2O_5 (Figure S5b-1) with a Mo doping concentration of 2.33 %. Similarly, the H-Ta_2O_5 crystal structure was constructed by substituting the interlayer oxygen atoms with the H atoms to form the H-Ta_2O_5 nanosheets (Figure S5c-1).

Supporting Information 4

1. Experimental setup

Our experiment employed a femtosecond CPA laser system that generates laser pulses with 250 kHz repetition rate, 800 nm central wavelength, and 70 fs pulse duration. The generated laser beam was split into two. One beam was frequency doubled by BBO crystal to generate 400 nm laser pulses. The other beam was reflected by a retro-reflector on a motorized linear stage and then focused onto the sample surface as the probe beam. Methyl Violet (MV) crystals were stick on a sample holder. Pristine Ta_2O_5 powders and powders of Ta_2O_5 with absorbed MV molecular (noted as MV-Ta_2O_5) were stick on a BK7 glass using black tap and were pressed by another glass to make a flat surface. The power of the pump pulse was 0.1mW, and 0.05mW for probe pulse, the corresponding fluence was 3.18 mJ/cm^2 and 1.59 mJ/cm^2, respectively.

2. **Results and Discussion**

Both pure Ta_2O_5 and MV-Ta_2O_5 samples were investigated to reveal the impact of photo-induced decay on the carrier dynamics behaviour in MV-Ta_2O_5. We measured the differential reflectivity $\Delta R/R_0$ of the probe beam as a function of the delay time between the pump and probe beams. This reflected the modification of the dielectric constant by the excited state carriers and lattice collective excitations along with the pump beam excitation.[17] In Figure S11, clear distinction between the dynamics of MV-Ta_2O_5 and pure Ta_2O_5 can be seen. Both amplitudes and lifetimes of the ultrafast dynamics are different for Ta_2O_5 and MV-Ta_2O_5. The relaxation feature of Ta_2O_5 is largely changed in the MV-Ta_2O_5 complex.

The experimental data of the ultrafast dynamics can be fitted by the following equation:

$$\frac{\Delta R}{R_0} = A_{fast} * \exp\left(-\frac{x}{\tau_{fast}}\right) + A_{slow} * \exp\left(-\frac{x}{\tau_{slow}}\right) + A_0, \qquad (4)$$

where A_{fast} and A_{slow} denoted amplitudes, τ_{fast} and τ_{slow} standed for the lifetimes of the excited carriers, A_0 was the background level. With Equation (4), we obtained the data analysis results of the dynamic features. The relaxation of pure Ta_2O_5 corresponded to a two-component exponential decay function (τ_{fast} =1.45ps, τ_{slow} =50.8ps) while the dynamics of MV-Ta_2O_5 corresponded to only one-component (τ_M =22.18ps).

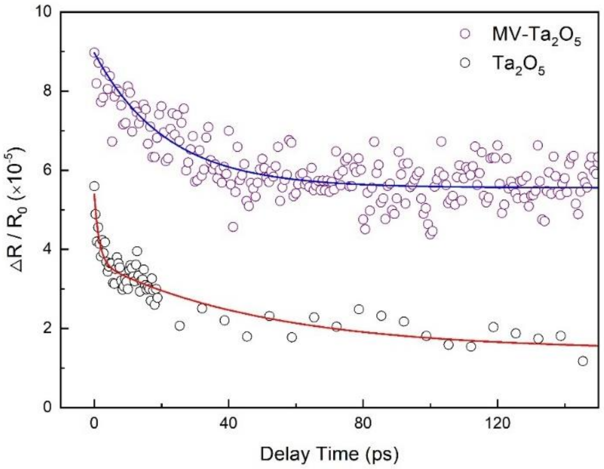

Figure S11. The carrier dynamics of Ta_2O_5 (black hollow circles) and MV-Ta_2O_5 (violet hollow circles) were collected under 0.1mW laser pump. Ta_2O_5 corresponded to a two-component exponential decay function (τ_{fast} =1.45ps, τ_{slow} =50.8ps, marked in red solid curve), and MV-Ta_2O_5 corresponded to a single component exponential decay function (τ_M =22.18ps, marked in blue solid curve). The temporal step sizes were 0.33 ps for the −5 ~ 20 ps delay time range and 3.33 ps for the 20 ~ 150 ps range. The pump laser power used was 0.1mW.

We furthermore performed a laser power dependence experiment on pure MV sample, for which we illustrated the results in Figure S12. The differential reflectivity of three MV crystals were collected under 0.02 mW, 0.05 mW and 0.1 mW pump powers, respectively. All three tested MV samples were exposed to pump laser beam for 60 minutes. It can be seen that for the higher pump power case, the dynamics curve clearly exhibited an additional faster relaxation component, now with τ_{fast} =0.96ps and τ_{slow} =6ps. For the lower pump power case, there is only one relaxation component, for which the lifetime is much longer, as τ =200ps. We contemplate there are two channels that contribute to the photo-induced decay of the MV molecule in our experiment on MV-Ta_2O_5: (a) the photo-catalytic degradation of MV

molecule by Ta_2O_5 and (b) the direct photo-bleaching for the MV molecule (without Ta_2O_5). The two factors combine together to yield the overall photo-induced decay. The decay rate for both channels increases with the increasing illumination laser beam power. Considering this, the dynamic under low pump power in Figure S12 (yellow diamonds) is closer to the pure dynamic of MV molecule without photo-induced decay.

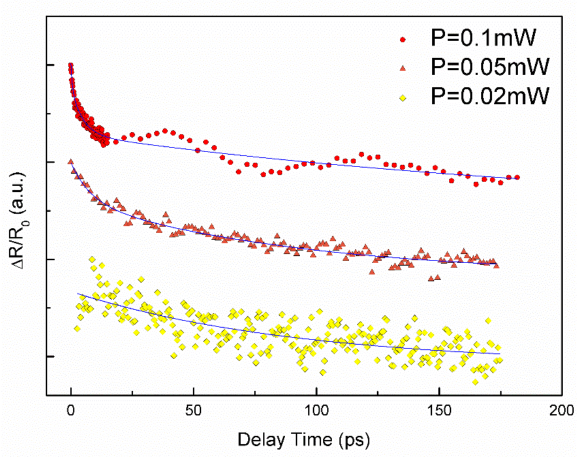

Figure S12. The dynamics of MV crystal under 0.02 mW (yellow diamonds), 0.05 mW (orange triangles) and 0.1 mW (red balls). A clear sharp decrease feature can be seen for MV samples under higher power. The blue solid lines denote fitting curves using exponential decay function. The temporal step sizes are 0.33 ps for the 0 ~ 20 ps range and 3.33 ps for the 20 ~ 175 ps range for P=0.1mW. The temporal step size for P=0.05 mW and 0.02 mW is 1 ps.

Table S4. Fitting Parameters of MV dynamics (P=0.1mW)

**Irradiated Time(min)**	**5**	**15**	**60**	**200**
τ_{slow} **(ps)**	**7**	**10**	**6**	**12**
τ_{fast} **(ps)**	**-**	**0.78**	**0.96**	**0.37**

References

[1] Q. Liu; L. Jiang; L. Guo. *Small* **2014,** *10*, 48-51.
[2] S. Cong; Y. Yuan; Z. Chen; J. Hou; M. Yang; Y. Su; Y. Zhang; L. Li; Q. Li; F. Geng; Z. Zhao. *Nat. Commun.* **2015,** *6*, 7800.
[3] D. Qi; L. Lu; L. Wang; J. Zhang. *J. Am. Chem. Soc.* **2014,** *136*, 9886-9.
[4] X. Wang; W. Shi; Z. Jin; W. Huang; J. Lin; G. Ma; S. Li; L. Guo. *Angew. Chem. Int. Ed. Engl.* **2017,** *56*, 9851-9855.
[5] Z. Zheng; S. Cong; W. Gong; J. Xuan; G. Li; W. Lu; F. Geng; Z. Zhao. *Nat. Commun.* **2017,** *8*, 1993.
[6] Y. Shan; Z. Zheng; J. Liu; Y. Yang; Z. Li; Z. Huang; D. Jiang. *npj Comp. Mater.* **2017,** *3*, 11.
[7] H. Wu; H. Wang; G. Li. *Analyst* **2017,** *142*, 326-335.
[8] A. Li; J. Lin; Z. Huang; X. Wang; L. Guo. *iScience* **2018,** *10*, 1-10.

[9] J. Zhang; Y. Pan; Y. Chen; H. Lu. *J. Mater. Chem. C* **2018,** *6*, 2216-2220.

[10] J. Lin; Y. Shang; X. Li; J. Yu; X. Wang; L. Guo. *Adv. Mater.* **2017,** *29*, 1604797.

[11] X. M. Lin; Y. Cui; Y. H. Xu; B. Ren; Z. Q. Tian. *Anal. Bioanal. Chem.* **2009,** *394*, 1729-45.

[12] W. B. Cai; B. Ren; X. Q. Li; C. X. She; F. M. Liu; X. W. Cai; Z. Q. Tian. *Surf. Sci.* **1998,** *406*, 9-22.

[13] P. Hohenberg; W. Kohn. *Phys. Rev. B* **1964,** *136*, B864-871.

[14] J. P. Perdew; K. Burke; M. Ernzerhof. *Phys. Rev. Lett.* **1996,** *77*, 3865-3868.

[15] H. J. Monkhorst; J. D. Pack. *Phys. Rev. B* **1976,** *13*, 5188-5192.

[16] P. J. Hay; W. R. Wadt. *J. Chem. Phys.* **1985,** *82*, 270-283.

[17] F. Sun; Q. Wu; Y. L. Wu; H. Zhao; C. J. Yi; Y. C. Tian; H. W. Liu; Y. G. Shi; H. Ding; X. Dai; P. Richard; J. Zhao. *Phys. Rev. B* **2017,** *95*, 235108.
